# A Self-Cleaning TiO_2_ Bacterial Cellulose Super-Hydrophilic Underwater Super-Oleophobic Composite Membrane for Efficient Oil–Water Separation

**DOI:** 10.3390/molecules28083396

**Published:** 2023-04-12

**Authors:** Yawen Cui, Xudong Zheng, Tongtong Xu, Biao Ji, Jinfeng Mei, Zhongyu Li

**Affiliations:** School of Environmental Science and Engineering, Changzhou University, Changzhou 213164, China

**Keywords:** bacterial cellulose, oil–water separation, self-cleaning, super-hydrophilic, membrane

## Abstract

Due to the increasingly serious problem of offshore oil spills, research related to oil–water separation has attracted more and more attention. Here, we prepared a super-hydrophilic/underwater super-oleophobic membrane (hereinafter referred to as BTA) using poly-dopamine (PDA) to adhesive TiO_2_ nanoparticles on the surface of bacterial cellulose, coated with sodium alienate by vacuum-assisted filtration technique. This demonstrates its excellent underwater super-oleophobic property. Its contact angle is about 153°. Remarkably, BTA has 99% separation efficiency. More importantly, BTA still showed excellent anti-pollution property under ultraviolet light after 20 cycles. BTA has the advantages of low cost, environmentally friendliness and good anti-fouling performance. We believe it can play an important role in dealing with problems related to oily wastewater.

## 1. Introduction

Water pollution is becoming an increasing concern [1,2]. The problems of oil leakage, oil exploitation and the transportation process, especially, will inevitably cause leakage due to improper management or some external objective reasons. Industrial oily wastewater is directly discharged due to untimely treatment or incomplete removal of oil droplets in oily wastewater, which can lead to leakage. Offshore oil spills and the discharge of industrial oily wastewater have caused great damage to the ecological environment. In addition, it seriously damages human safety and health. Non-degradable waste oil may produce different toxic elements, which will pollute the living environment and affect human health [3]. For example, the toxins contained in oily wastewater can affect the activity of human hormones and even cause cancer. In addition, oily wastewater contains a large number of bacteria, which can also damage human health. Traditional methods, which include centrifuges, sedimentation tanks, filtration and select flotation, which have relatively low separation efficiency and high cost. They also cause re-pollution to the environment [4,5]. Therefore, an urgent task is to invent new oil–water separation materials and improve the original technology to solve the problem of oil–water mixtures.

In recent years, superwetting materials have attracted more and more attention in oil–water separation due to their unique surface waterproof, self-cleaning and lubricating characteristics [6,7]. Inspired by this, super-hydrophobic materials have gained popularity [8]. Oxalic acid and sodium silicate are used to form a hydrophobic surface on the brick to separate the oil in the mixture from the water phase [9]. Shang et al. blended poly-dopamine with 1*H*,1*H*,2*H*,2*H*-perfluor-odecanethiol to prepare super-hydrophobic surfaces with excellent adsorption and separation ability [10]. However, these materials are easily contaminated by way of oil, or even irreparable damaged [11,12,13]. Because the nature of super-hydrophobic materials is to not let water pass through themselves, they let oil droplets pass through the material. This will cause oil droplets to contaminate the surface or internal pores of the super-hydrophobic material, which is not conducive to secondary use, thus affecting the service life of the material itself. In summary, it is worth citing that super-hydrophilic/underwater super-oleophobic substances can be used for oil–water separation. This wetting material with hydrophilic and oleophobic properties is of increasing interest [14,15]. Liu et al. used PDA as an adhesive and loaded starch and polyimide at the same time to prepare a net with super-hydrophilic underwater super-oleophobia [16]. These materials allow water to penetrate through, but repel oil droplets with larger particle sizes, thus protecting the membrane from oil droplets. The biggest advantage of hydrophilic materials is to ensure that the material can maximize the service life of the material in experimental or practical application and improve the reusability of the material. For example, Cheng et al. prepared an underwater super-oleophobic separation membrane by combining HS(CH_2_)_11_OH. This separation membrane has high separation efficiency in oil–water separation [17]. Xu et al. summarized the structural characteristics of natural polyphenol–matrix composites, especially the application of nanofibers and membranes in water treatment [18]. In Zhu et al., Cu(OH)_2_ nanoparticles were loaded on the surface to prepare metal fiber membranes, and the metal fiber membrane has better separation efficiency in oil–water separation [19]. Although hydrophilic and oleophobic materials have good antifouling properties, their high cost and toxicity limit their application [20]. Since in the repeated oil–water separation test, the sample membrane will come into contact with the oil–water mixture during the filtration process, some oil droplets will remain on the surface of the membrane after the test, and the sample membrane will inevitably be contaminated. This significantly reduces the service life of the membrane material. Consequently, it is important to prepare and research non-toxic, cheap and effective anti-fouling membrane materials [21].

Alginate is extracted from brown algae. In addition, it has the characteristics of being renewable, relatively low cost and harmless to the environment. As an amply available bio-polymer with low toxicity, it has been extensively studied for many years for an extensive variety of applications, specifically on the surface of tissue, osmotic evaporation and membrane separation [22,23,24]. Because alginate shows swelling and degradation properties in water, it is somewhat limited in its use. Recent studies have proved that alginate has good alkali resistance, so it can play an important role in offshore oil leakage, and also has underwater super-oleophobic characteristics. This can play a key role in the treatment of oily wastewater [25]. In addition, sodium alginate is used as a functional monomer in the membrane-making process, and it can also corrode membranes with dense pore sizes into porous structures. It can expand the pore size of the membrane, increase the water flux of the hydrophilic membrane and further improve the separation efficiency of the hydrophilic membrane for the oil–water mixture. However, after performing oil–water separation many times, the material will inevitably be contaminated by oil droplets, so a substance is needed to prevent such contamination.

Titanium dioxide (TiO_2_) itself has the advantages of environmental protection, non-toxicity and low price, it is widely used in many fields [26,27], such as photo-catalytics, solar batteries [28] and many more. Typically, titanium dioxide is used as a catalyst to degrade organic pollutants under UV light, because titanium dioxide has strong activity under ultraviolet light. In recent years, titanium dioxide is increasingly used to solve problems related to oily wastewater because it can decompose organic pollutants under ultraviolet light [29,30]. It is simple and convenient to use titanium dioxide to remove some dyes and antibiotics that may be mixed in oily wastewater. Therefore, with this superior performance, a membrane loaded with titanium dioxide has a great improvement in the anti-pollution ability of the membrane. This extends the service life of the membrane for the recycling of the membrane. Zhang et al. prepared an underwater super-oleophobic membrane with great self-cleaning ability by using sodium silicate and titanium dioxide nanoparticles [31]. Therefore, it is of great significance to explore lower cost and easier preparation of TiO_2_ membrane materials. In order for titanium dioxide nanoparticles to stick tightly to the surface of the composite, an adhesive is needed.

**Scheme 1:**
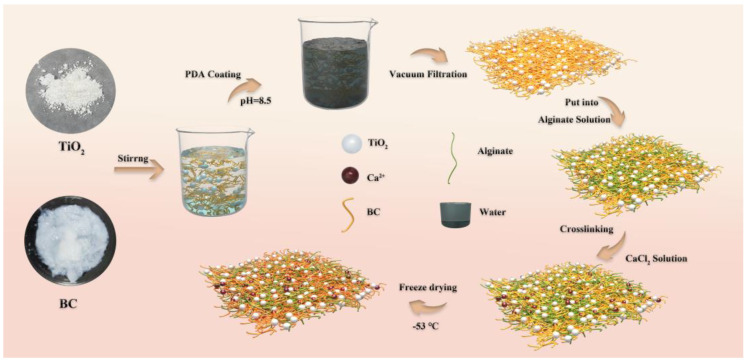
Synthesis path diagram of BTA.

As an adhesive, dopamine plays the role of adhesive carrier in membrane synthesis and has been increasingly used in the film-making process [32]. Wang et al. deposited a mixture of polydopamine and Ca on the net to improve the microstructure of the net surface and prepared an underwater super-oleophobic oil–water separation net with excellent mechanical properties [33]. Yang et al. prepared a water-treatment membrane (PDA/TOB@CA) with synergistic antibacterial properties of polydopamine nanoparticles doped with tobramycin using cellulose as the base [34]. It is worth mentioning that poly-dopamine (PDA) acts as an adhesive on the surface of the membrane, making the membrane stick closely to the supporting substance. Gluing dopamine to the surface of the membrane does not change the chemistry and internal structure of the membrane itself. Since dopamine is a hydrophilic substance, it can improve its hydrophilic properties, increase water flux and improve its separation efficiency for hydrophilic oleophobic membranes. Due to the rich conjugated P-structure in the PDA network, it has good light absorption and electron transport performance, which will be beneficial to the photo-catalytic reaction [35]. Loading dopamine and TiO_2_ nanoparticles on the membrane not only promoted the hydrophilicity of the membrane, but also showed positive anti-pollution performance through the ability of TiO_2_ to decompose organic pollutants under ultraviolet light. Firstly, TiO_2_ nanoparticles smaller than 200 Å have incomplete coordination wires of surface metal atoms. Therefore it shows high affinity for oxygenated ligands. Because the surface of the BC membrane is loaded with dopamine, dopamine adheres titanium dioxide particles tightly to the surface of the membrane through the coordinated action with Ti atoms. Secondly, the hydroxyl group on the surface of titanium dioxide binds to the amino group contained in dopamine to form a stable ammonia bond on the bacterial fiber. Yang et al. prepared super-hydrophilic, underwater super-oleophobic aerogels by a one-pot polycondensation method of 5,6-dihydroxyindol and formaldehyde. The aerogel not only has good oil–water separation performance, but also can remove organic dyes and heavy metal ion pollutants in wastewater at the same time [36]. In this article, a low-budget, easy-to-prepare super-hydrophilic/underwater super-oleophobic bacterial fiber membrane (BTA) was investigated which can decompose organic pollutants under ultraviolet light and has excellent anti-pollution performance. It was made by cross-linking TiO_2_ nanoparticles and sodium alginate, which were immobilized on the bacterial cellulose membrane surface by way of dopamine, with the aid of ionic cross-linking and freeze-drying technique. BTA separates both low- and high-viscosity oil–water mixtures with a separation efficiency of more than 99%. It is worth noting that the BTA has excellent stability and heat resistance. After 20 cycle experiments, a separation efficiency of more than 99% was maintained, indicating that the composite membrane had good reusability. In addition, BTAs maintain their excellent UV-driven self-cleaning properties even after contamination with organic solvents. BTA not only has excellent separation efficiency and water flux for oil–water mixtures, but also has self-cleaning properties induced by ultraviolet light. This kind of membrane material with excellent performance has great development prospects in oil–water separation. The artificial synthesis of BTA is outlined in Scheme 1.

## 2. Results and Discussion

### 2.1. Preparation and Structural Study of BTA

**Figure 1:**
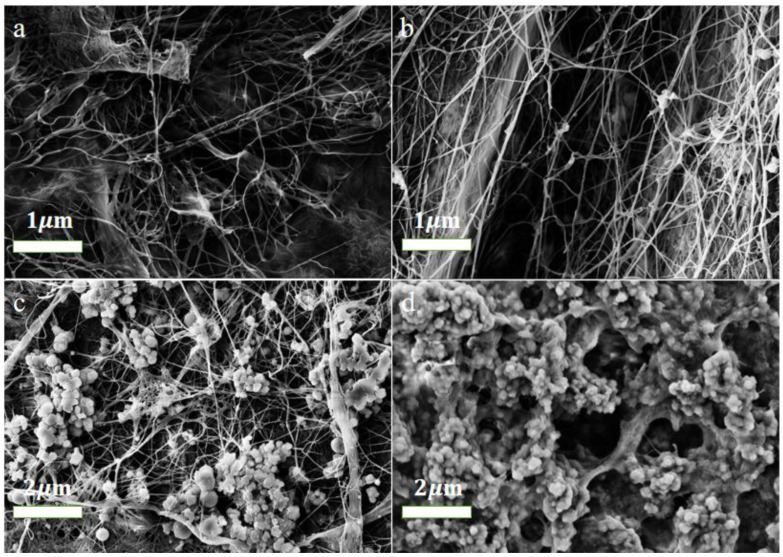
(**a**) SEM images of BC membrane. (**b**) BC adhesive PDA composite membrane. (**c**) TiO_2_ composite membrane was supported by BC adhesive PDA and (**d**) BTA.

The morphology of the original BC membrane and other three composite membranes were analyzed by SEM. Figure 1a shows the three-dimensional, extensively interwoven nanometer shape of the pristine bacterial cellulose, with a fiber diameter of about 30 nm. After coating the PDA layer, the diameter of these fibers efficiently increases to approximately 90 nm, demonstrating the effect of poly-dopamine on the thickness of the membrane, as viewed in the BC/PDA sample in Figure 1b. We can also see that the PDA coating is uniformly loaded on the BC fiber, which facilitates subsequent loading of other substances. The BC/PDA/TiO_2_ sample in Figure 1c actually shows the distribution of TiO_2_ nanoparticles along the fiber. Titanium dioxide particles aggregate on the surface of the membrane and are uniformly loaded on the BC fibers, constructing a micro–nano structure which is indispensable for the hydrophilicity of the membrane. Due to the large specific surface area, nano-TiO_2_ has stronger UV absorption capacity, so it has stronger photocatalytic ability. In the case of the BTA, Figure 1d indicates that the 3D mesh structure was not destroyed, and that the surface of the composite membrane has a honeycomb porous morphology with a random distribution of pores after the addition of SA. These holes can effectively help us separate oil and water.

**Figure 2:**
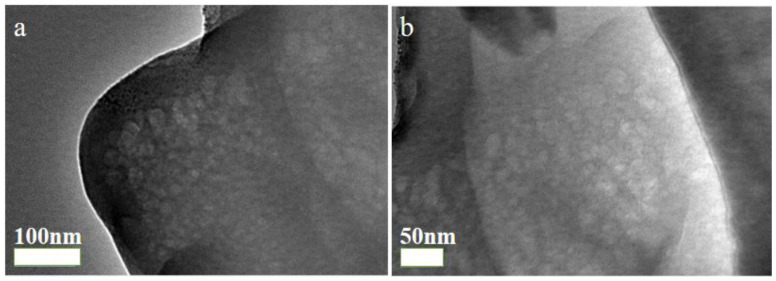
(**a**) TEM images of BTA and (**b**) BTA after magnification.

This is shown in Figure 2, transmission electron microscopy analyzed the topography of the BTA. It can be seen that the surface of the membrane has a clear pore structure. Scanning electron microscopy characterization of BTA was also confirmed. 

**Figure 3:**
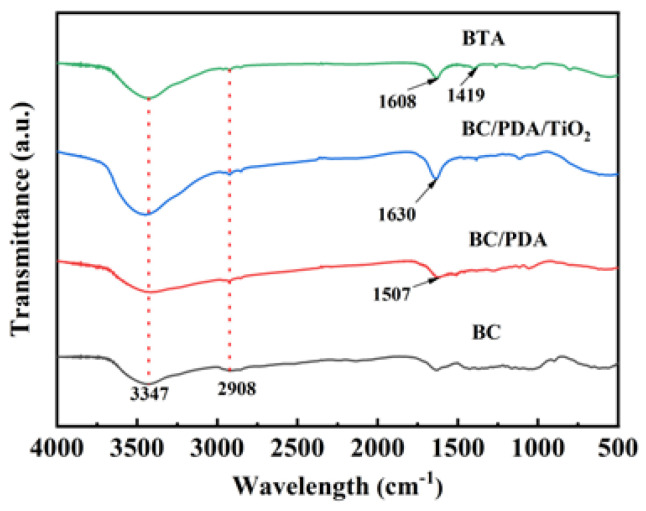
FT−IR images of four different load membranes.

As shown in Figure 3, the chemical structures of the original BC membrane and the other three composite membranes were analyzed by FT-IR. The stretching vibrations of the O-H, CH_2_-CH, and C-O bonds, respectively, are represented by the adsorption peaks of the original BC membrane at 3347, 2908 and 1050 cm^−1^. The intensity of these peaks decreased after the BC membrane was loaded with PDA and further decreased after the TiO_2_ was loaded. The adsorption height at 1507 cm^−1^ in BC/PDA corresponds to the C-N stretching vibration. After the BC membrane was loaded with PDA and TiO_2_, the absorption peak of the sample membrane at 1630 cm^−1^ corresponds to the telescopic vibration of Ti-O [37]. With respect to asymmetric and symmetric -COO- stretching vibrations and -OH stretching, respectively, BTA exhibits unusual peaks at 1608 cm^−1^ and 1419 cm^−1^. It also has a broader adsorption band for -OH stretching at 3364 cm^−1^ [38].

**Figure 4:**
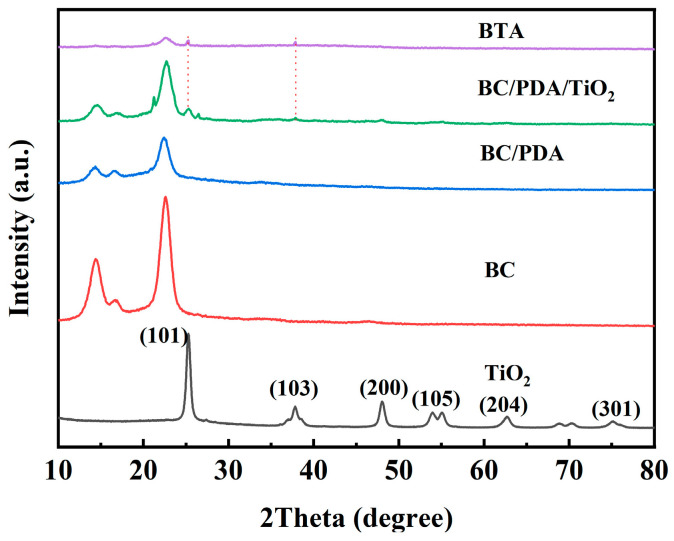
XRD images of four different load membranes.

Figure 4 shows the crystal structure of the original BC membrane and the other three composite membranes using XRD. Typical peaks of crystalline cellulose at 14.5° and 22.3° can be considered in each the BC and BC/PDA samples, and these peaks were obviously shielded after TiO_2_ loading and SA loading, probably due to the coating of the nanometer surface [39]. In the BTA sample, 25.31°, 37.90° and 48.02° correspond to the (101), (103) and (200) planes of the crystal diffraction anatase TiO_2_, respectively (JCPDS No. 21-1272). In BTA samples, sodium alginate does not exhibit diffraction peaks in XRD. In addition, due to the secondary loading of sodium alginate on the basis of titanium dioxide, the peak strength of titanium dioxide is greatly weakened.

**Figure 5:**
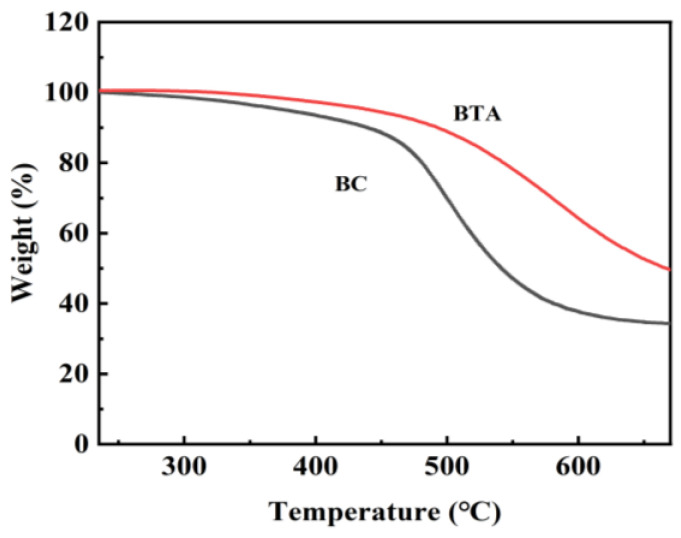
TGA images of BC and BTA.

We analyzed the thermal stability of the original BC membrane and the composite BTA with thermogravimetry (TGA). Figure 5 shows the thermogravimetric curves of BC membrane and BTA. BC membrane mass loss suddenly becomes greater at 450 °C because BC is a biomass material that quickly loses weight and carbonizes at high temperatures. In contrast, after loading PDA and TiO_2_, the thermal stability of BTA is significantly improved due to the heat resistance of dopamine and the coordination of metal Ti. This also proves that BTA has good thermal stability.

### 2.2. UV Wettability of the BTA

**Figure 6:**
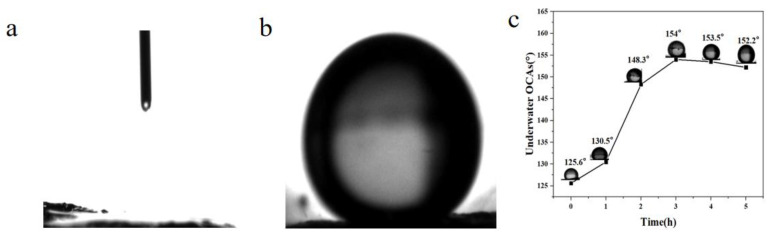
(**a**) Contact angle image of water is 0°. (**b**) Underwater oil contact angle is 156.5°. (**c**) The change of oil contact angle under UV light in 0~5 h.

In the treatment of oil–water mixtures, the wettability of the membrane is one of the important influencing factors. Water contact Angle (WCA) in air and underwater oil contact Angle (UOCA) measurements were made to demonstrate the composite membrane’s wettability. Figure 6a shows that the WCA of the BTA is 0° and the UOCA of the BTA membrane is 156.5°. In Figure 6b, it can be seen that PDA, titanium dioxide and sodium alginate have a function in the membrane’s WCA and UOCA. The BTA had a WCA of 0°, indicating that the membranes had super-hydrophilic properties. The BTA had a UOCA of 156.5°, indicating that the membranes had underwater super-oleophobic properties. Figure 6c shows the oil contact change of the BTA below water for specific UV irradiation times. Although the BTA has good underwater super-oleophobic, and UOCA value is 156.5°, but after repeated experiments, the contact angle becomes 125.6° after the surface is contaminated by oil droplets. After 5 h of ultraviolet light irradiation, the composite membrane is placed in a closed space. The value of the UOCA of the composite membrane increases from 125.6° to 154°, and tends to be stable after 3 h, indicating that the composite membrane changes from underwater oleophobic to underwater super-oleophobic under ultraviolet light. This shows that BTA has super-oleophobic properties in UV induction.

### 2.3. Oil–Water Separation Tests

**Figure 7:**
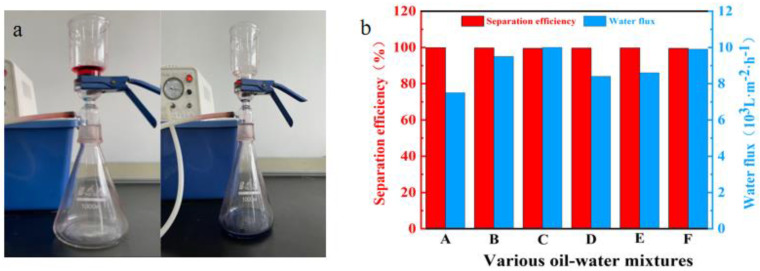
(**a**) Dynamic process of oil−water separation (**b**) Water flux and separation efficiency of six oil−water mixtures (A−F represent dichloroethane, petroleum ether, toluene, soybean oil, diesel, hexane and water mixtures, respectively).

Figure 7a indicates the apparatus used to separate the mixture of hexane−water. As shown in Figure 7a, the oil–water mixture with a ratio of 1:1 is poured into a flask above the vacuum filtration equipment, the peristaltic pump is turned on and it is found that water rapidly flows through the composite membrane into the bottom flask, while the oil is blocked in the upper part without the membrane. As shown in Figure 7b, the effects show that BTAs have high separation efficiencies of over 99%. In addition, the water fluxes of six kinds of oil–water combinations have been measured: the water flux values of the three oil–water mixtures A, D and E are 7428 to 8774 L·m^−2^h^−1^, showing lower water fluxes because these three oils are heavy oils and the viscosity is high. The water flux value of the three oil–water mixtures B, C and F is 9597–10,000 L·m^−2^h^−1^, which shows a higher water flux. Because the other three oils are light oils, the viscosity is small. Figure 7b shows the flux and separation efficiency of the composite membrane for three heavy oils and three light oil mixtures. Through the above analysis and test, the separation efficiency of the BTA for heavy oil and light oil has reached more than 99%, which has a certain reference for dealing with complex oil–water separation problems in the future.

**Figure 8:**
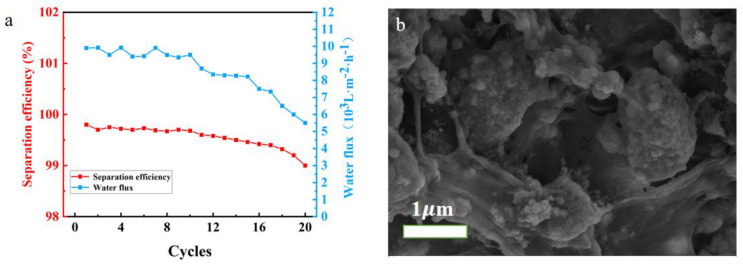
(**a**) Changes in water flux and separation efficiency over 20 cycles. (**b**) SEM image of BTA after 20 oil−water separation cycles.

The sustainable usability of the membrane is also one of the influences on the evaluation of the performance of functional membranes. Figure 8a shows the test effect of the BTA over 20 cycles. The effect remained high for the first 10 cycles with no noticeable change. In the following 10 cycles, the effect of both aspects of the BTA began to gradually decrease, which may be because the composite membrane was blocked by oil droplets after several experiments. Shown in Figure 8b, the microstructure of BTA used to be determined by SEM, and the oil droplets blocked the pore size of the membrane after 20 oil–water separation cycles. This indicates that oil droplet contamination is the reason for the decrease of separation efficiency and flux of the composite membrane. However, it does not change the structure of BC: the 3D mesh structure was not damaged and the TiO_2_ nanoparticles remained firmly attached to the BC fiber. In addition, the stability of BTA can be explained by the fact that the XRD comparison before and after the S1 cycle is basically unchanged (Figure S1).

### 2.4. Self-Cleaning Performance of the BTA

**Figure 9:**
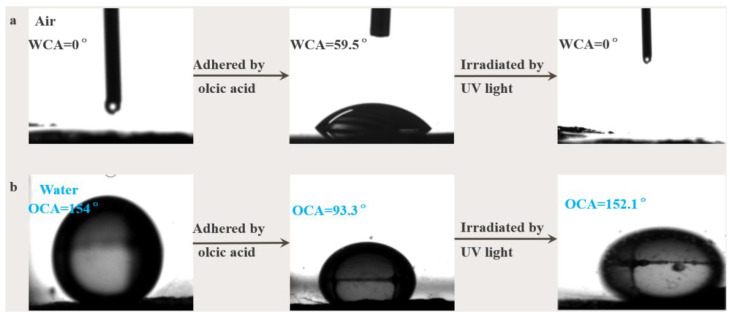
(**a**) The change of water contact angle (WCA) of the BTA by UV light. (**b**) The change of oil contact angle (UOCA) of the BTA by UV light.

Conventional membranes have excessive surface energy and are easy to be polluted in oil–water separation. In long-term use, the surface of the membrane will inevitably be polluted or blocked by oil droplets, affecting the continued use, so the use of self-cleaning performance of the membrane from the long-term perspective is very meaningful. TiO_2_ is characterized by degradation of organic matter in ultraviolet light and allows BTA to have appropriate anti-fouling and self-cleaning properties. TiO_2_ nanoparticles have unique photo-induced super-hydrophilic properties that endow them with outstanding performance in self-cleaning coatings and functional interfacial materials with special wettability. After ultraviolet (UV) irradiation, the surface of TiO_2_ produced microstructures composed of hydrophilic and oleophilic phases, resulting in a super-hydrophilic property. Figure 9 shows the process of restoring the contact angle of BTA through ultraviolet irradiation, thus proving that BTA exhibits self-cleaning. Using low volatile oleic acid as a model oil, the WCA in air multiplied from 0° to 59.5°, the results showed that the composite membrane changed from super-hydrophilic membrane to hydrophilic membrane because oil droplets will remain on the surface of the membrane during the test. At the same time, the UOCA of the BTA decreases from 156.5° to 83.4°. It is proved that residual oil droplets adhere to the membrane and contaminate the surface of the membrane. Therefore, the BTA lost its original wettability and could not continue to separate the oil–water mixtures.

The WCA of the membranes contaminated with oleic acid lowered to 0° and the value of the UOCA elevated to 152.9° after 12 h of UV irradiation. These outcomes show that the BTA can, without problems, recover their super-hydrophilic and underwater super-oleophobic properties after UV irradiation. The BTA is proven to have self-cleaning properties. It is nicely acknowledged that anatase type titanium dioxide is generally considered to be the most photo-active crystalline form. Under UV irradiation, TiO_2_ particles loaded by the composite membrane decompose the residual oil droplets on the membrane surface into hydrophilic compounds. By this method, the oil droplets remaining on the membrane surface are resolved and restore the membrane’s super-hydrophilic and underwater super-oleophobic properties.

## 3. Materials and Methods

### 3.1. Materials

Bacterial cellulose (BC) was bought from Guilin Qi Hong Technology Co. TiO_2_, dopamine hydrochloride (DA, 98%), trimethyl aminomethane (tris, 99.99%), methylene blue (MB) and Sudan red III (AR) were bought from Sinopharm Chemical Reagent Co., Ltd. (Shanghai, China). Sodium alienate (SA) (sodium salt of confidant) was bought from Aladdin reagent (China). Soybean oil was bought from local supermarkets. Diesel was bought from local gas stations. Dichloroethane (≥99%), petroleum ether (≥99.5%), toluene (≥99.5%) and hexane (≥99.5%) were obtained from Sinopharm Group (China). All of the above reagents, as well as ethanol, were analytical grade.

### 3.2. Synthesized Composite TiO_2_ Membrane

On the basis of BC and TiO_2_, through a simple vacuum-assisted filtration process, the composite PDA adhesive forms a composite membrane. First, an ultrasonic cleaner was used to pour titanium dioxide nanoparticles (0.04 g) into distilled water (50 mL) for 10 min. Then it was placed on a magnetic stirrer and stirred for 10 min. At the same time, the BC membrane (0.3 g) was evenly broken with a homogenizer, added to distilled water (100 mL) and stirred for 20 min. Then the evenly stirred titanium dioxide solution was poured into the BC solution and stirred for 1 h with a magnetic stirrer to mix it well. Afterwards, the pre-configured tris(hydrometry) amino-methane (0.2 g) and dopamine (0.46 g) mixed solution were taken out. Then a mixed solution of BC and titanium dioxide was poured into it and stirred for 6 h. Finally, the mixed solution was filtered through suction in a vacuum filter to obtain a preliminary composite TiO_2_ membrane, and then finally a composite membrane was obtained after freeze-drying for 24 h. Poly-dopamine was uniformly loaded on the surface of the BC membrane, and nanometer TiO_2_ particles were uniformly loaded on the BC fibers.

### 3.3. Preparation of BTA Membrane

BTA was prepared by way of a simple magnetic stirring process. First, 100 mL of distilled water was mixed with sodium alginate (SA) and stirred for 6 h. Then the BTA was positioned in the above mixed answer and stirred for 12 h. The BTA was then placed in a CaCl_2_ (4 wt%) solution and stirred magnetically for 24 h. In order to remove substances that are unsaturated or unfirmly loaded on the surface of the composite membrane, the surface of the composite membrane was repeatedly rinsed with distilled water. Then the rinsed composite membrane was placed into a clean tray and frozen in the refrigerator overnight, after which the membrane was placed in a freeze dryer for 24 h, and finally BTA was obtained.

### 3.4. Oil–Water Separation Experiments

After water wetting, the composite membrane was clamped between two volumetric bottles with a diameter of 0.5 cm and connected with a peristaltic pump, thus forming a simple oil–water separation installation. The preparation method of oil–water mixture is as follows: 30 mL oil was dyed with Sudan red-III and 30 mL water with methylene blue, and then poured into 100 mL beaker. For the oil–water separation experiment, the peristaltic pump was opened, and the mixture was passed through the oil–water separation device until it was completely separated. The water quality was weighed after separation. In the experiment, hexane was used as an example. The following is the separation efficiency (η) calculation formula (1):

(1) $$\eta =\frac{m_{1}}{m_{0}\;}\times 100\%$$

where m_0_ (g) is the initial quantity and m_1_ (g) is the quantity at t time.

The calculation formula of flux is (2):

(2) $$J=\frac{V}{S\Delta t}$$

where J (L·m^−2^·h^−1^) is flux, V (L) is the total amount of water passing through the membrane over a period of time, S (m^2^) is the actual area of the membrane sandwiched between the suction bottles and Δt (h) is the actual time it takes for water to pass through the membrane and oil to be trapped at the top of the membrane.

The effectiveness of BTA was once confirmed by means of 20 oil–water separation experiments. After 20 UV drives, the membrane after oil–water separation had good anti-fouling properties. 

### 3.5. Self-Cleaning of the Membrane

To test the anti-contamination performance of BTA against organic pollutants, the membranes have been first organically contaminated with 5 wt% oleic acid in an ethanol solution. The contact angle of the composite membrane was measured with a contact angle tester for water contact angle and underwater oil contact angle. The wettability of the membranes was once again characterized by way of contact angle measurements after irradiating the membranes under a UV lamp (power: 15 W × 12; wavelength: 365 nm) in a fully enclosed black container (UV-3000 analyses, Japan Technology Ltd., China).

## 4. Conclusions

UV-induced self-cleaning BTAs have been successfully prepared by easy vacuum-assisted filtration of suspensions containing TiO_2_ nanoparticles. The prepared composite membrane has hierarchical structure, nano-porous morphology and super-hydrophilic/underwater super-oleophobic surface. It can effectively separate heavy oil and light oil mixture and has high separation efficiency. Importantly, BTA shows significantly increased water flux and underwater oil contact angle under UV irradiation, which gives it promising development potential in oil–water separation. The photo-catalytic degradation of contaminants with the aid of anatase-type TiO_2_ results in good self-cleaning overall performance of the produced membranes after oil contamination. In this paper, a multifunctional oil–water separation membrane method is proposed by simple vacuum filtration and freeze-drying techniques.

## Data Availability

Our research data will be available on demand.

## Supplementary Materials

The following supporting information can be downloaded at: https://www.mdpi.com/article/10.3390/molecules28083396/s1, Figure S1: BC membrane and XRD of BTA before and after 20 cycle experiments.
