# Supplementary material for: A Self-Cleaning TiO2 Bacterial Cellulose Super-Hydrophilic Underwater Super-Oleophobic Composite Membrane for Efficient Oil–Water Separation
Source: Molecules. 2023 Apr 12;28(8):3396. doi: 10.3390/molecules28083396 (PMC10141678; doi:10.3390/molecules28083396)
Supplement: Supplementary file 1 [file molecules-28-03396-s001.zip › molecules-2267033-supplementary.pdf]

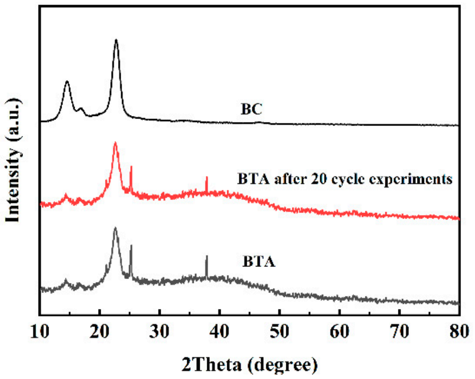

Figure S1. BC membrane and XRD of BTA before and after 20 cycle experiments

Figure S1 shows that the coordinated reaction of the sediment layer with the substrate BC membrane can be seen by the XRD of BTA before and after BC and separation experiments. By comparing XRD before and after the BTA cycle test, it was found that the load was still firmly adhered to the substrate BC membrane, which proved that the deposition layer was stable.
